# First person – Elia Escasany

**DOI:** 10.1242/dmm.049224

**Published:** 2021-10-01

## Abstract

First Person is a series of interviews with the first authors of a selection of papers published in Disease Models & Mechanisms, helping early-career researchers promote themselves alongside their papers. Elia Escasany is first author on ‘ Transforming growth factor β3 deficiency promotes defective lipid metabolism and fibrosis in murine kidney’, published in DMM. Elia is a PhD student in the lab of Gema Medina-Gómez at Universidad Rey Juan Carlos, Madrid, Spain, investigating the role of TGFβ in renal fibrosis.


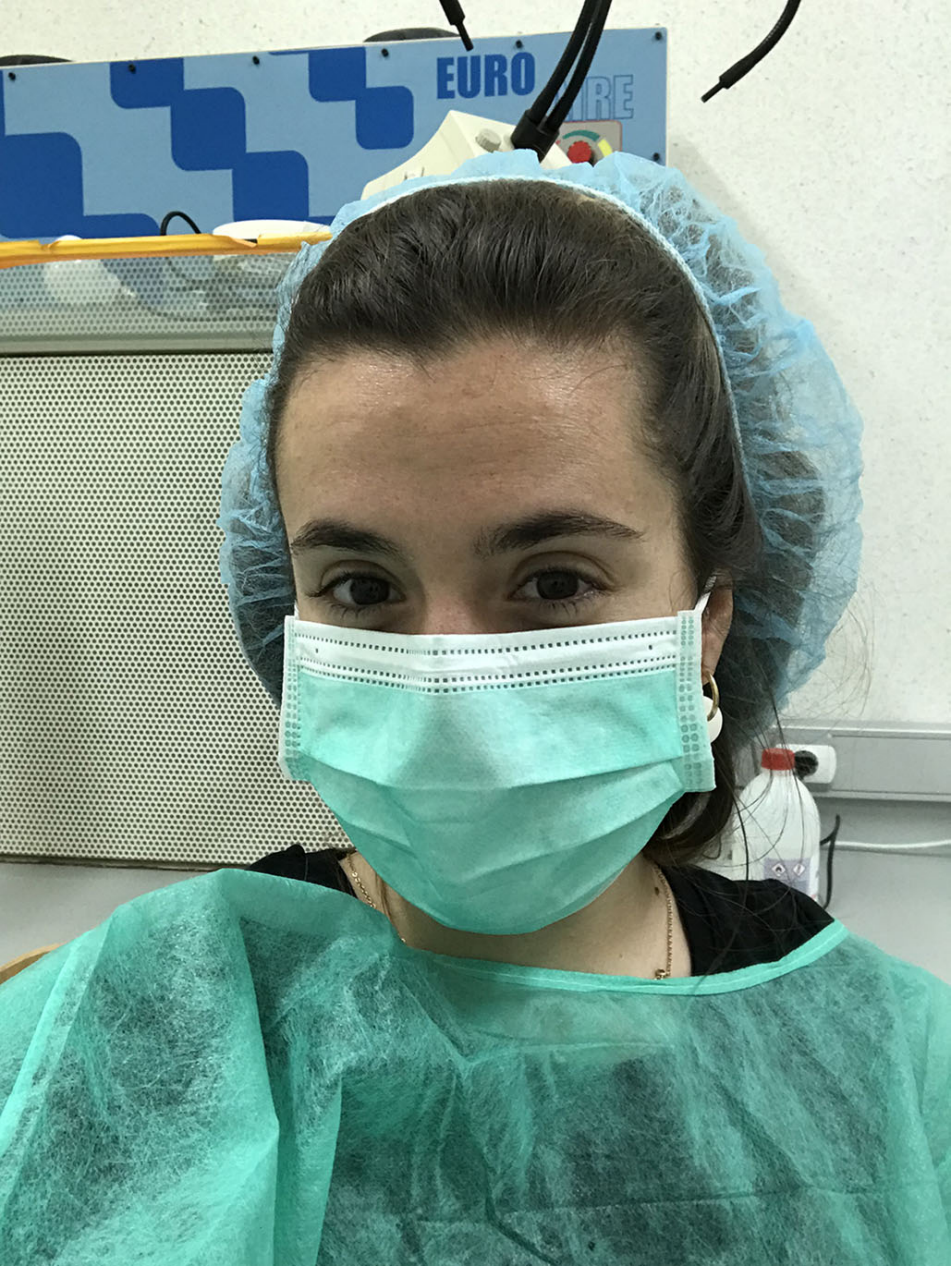


**Elia Escasany**

**How would you explain the main findings of your paper to non-scientific family and friends?**

Chronic renal diseases (CKDs) affect around 10% of the world population and currently have limited treatments and no cure. When a patient develops CKD, there is nothing doctors can do to revert it or even stop it from worsening. At present, what CKD treatments do is slow down the disease progression. As a result of this, CKD is the only disease for which the mortality rate has continued to increase in the last years. Moreover, CKD is an important cause of cardiovascular diseases and deaths. That is why it is crucial to understand in detail the mechanisms leading to CKD to make an early diagnosis and develop more-effective treatments. CKDs involve many renal diseases with different causes. However, what all CKDs have in common is the presence of fibrosis. Fibrosis is the way scars form inside the body, and it is an interesting target for treatments since it would benefit all CKD patients, regardless of the original cause of the renal disease. What I have been working on for the past 5 years is understanding the role of the main family of proteins involved in renal fibrosis, TGFβ.

**What are the potential implications of these results for your field of research?**

The TGFβ family involves three isoforms: TGFβ1, TGFβ2 and TGFβ3. For a long time, the only isoform that was studied was TGFβ1, and it was assumed that the two other isoforms behaved in a similar manner. In this paper, we discovered that TGFβ3 and TGFβ1 do not have redundant functions and that the lack of TGFβ3 leads to renal disease. Moreover, it seems like the relative amount of the different isoforms is key for the physiological function of the kidney. I think that this could explain the lack of success in the use of non-isoform-specific TGFβ antibodies as therapies and may promote more research in the field of fibrosis. I also believe that these results could have an important implication in the understanding of other fibrotic diseases, as fibrotic processes seem to be similar and involve the TGFβ family in many organs.

“[…] whole-body heterozygous models are the best approach to mimic what really happens in real human patients.”

**What are the main advantages and drawbacks of the model system you have used as it relates to the disease you are investigating?**

I have been working with a whole-body heterozygous TGFβ3-knockout mouse model. This means that the animals lack only one of the two alleles (50%) that encode for the TGFβ3 protein in all their organs. A drawback of using a heterozygous model is that the disease expresses in a more subtle way so it is more difficult to observe statistically significant changes. A drawback of using a whole-body model to study only one organ (the kidney in this case) is that sometimes it is not easy to know if the phenotype you are observing in your organ of interest in due to the lack of the gene you have knocked out or a secondary effect of the malfunctioning of another organ. However, whole-body heterozygous models are the best approach to mimic what really happens in real human patients.

**Figure:**
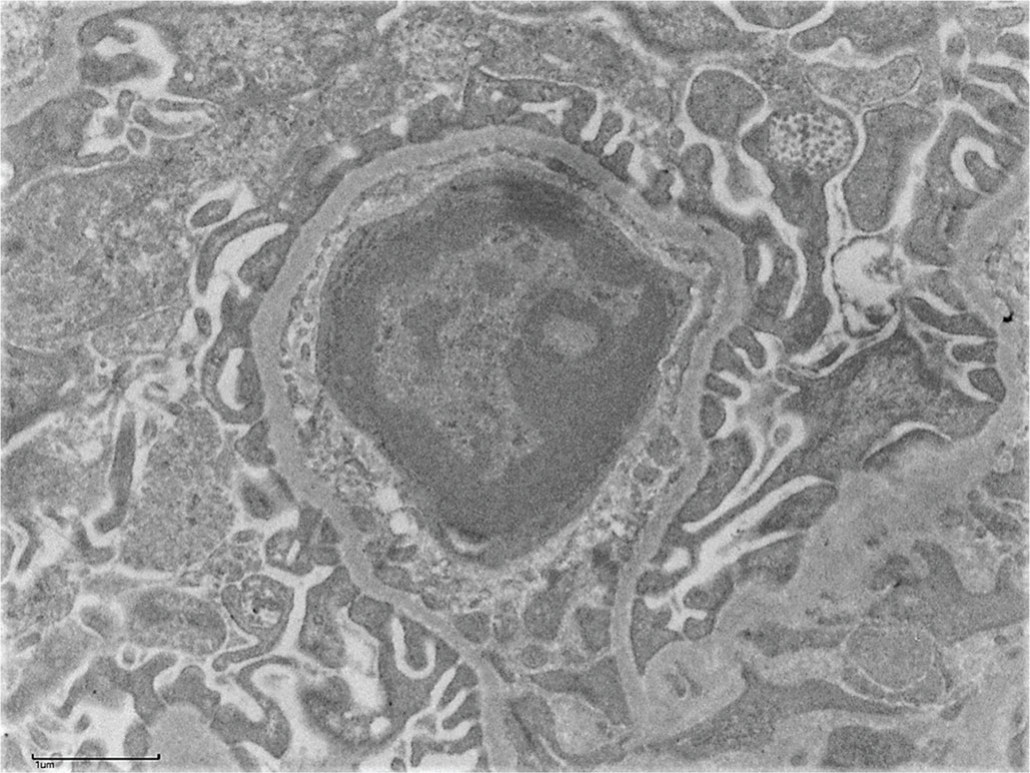
An injured glomerulus of a mouse lacking TGFβ3 observed through an electron microscope.

**What has surprised you the most while conducting your research?**

When I was analysing the activation of the downstream pathways of the TGFβ receptors, I expected to observe a decrease in the basal activation levels in the mice lacking TGFβ3 or maybe observe no changes. However, I was very surprised to observe an overactivation of the non-canonical pathways in our TGFβ3-knockout mouse model. This result prompted me to look deeper into the mechanisms and trigger the mechanistic part of this paper.

**Describe what you think is the most significant challenge impacting your research at this time and how will this be addressed over the next 10 years?**

I think it would be interesting to look deeper into the mechanisms connecting TGFβ3 with the other isoforms and the activation of the downstream pathways. Moreover, it would be interesting to generate cell-specific knockout *in vitro* and *in vivo* models to evaluate the role of TGFβ3 in the different renal cell types.

“[…] PhD students are responsible for an important part of the scientific content that is produced worldwide. However, we are not paid or treated accordingly […]”

**What changes do you think could improve the professional lives of early-career scientists?**

I think that, as a PhD student, which is the first step of your scientific career, you feel a lot of pressure with the deadlines. In order to meet those tight deadlines, you have to put in a lot of extra hours of work. Especially when you work with animals, you sometimes have to work on weekends. This lack of rest and time off to spend with your close ones, together with the stress and pressure, takes its toll on the mental health, and even physical health, of many PhD students. Moreover, PhD students are responsible for an important part of the scientific content that is produced worldwide. However, we are not paid or treated accordingly, and we have to apply for funding regularly. I believe we need to put more emphasis on the reconciliation of work and private life as well as re-evaluate the job stability and salaries/grants.

**What's next for you?**

Firstly, I have to complete my PhD, and, after that, I hope to continue making science that is useful and has an impact on the quality of life of patients.
